# The incidence of infantile hypertrophic pyloric stenosis nearly halved from 2005 to 2017: analysis of German administrative data

**DOI:** 10.1007/s00383-020-04810-0

**Published:** 2021-01-20

**Authors:** Christina Oetzmann von Sochaczewski, Oliver J. Muensterer

**Affiliations:** 1 Klinik und Poliklinik für Kinderchirurgie, Universitätsmedizin der Johannes-Gutenberg-Universität Mainz, Langenbeckstraße 1, 55131 Mainz, Germany; 2 Sektion Kinderchirurgie der Klinik für Allgemein-, Viszeral-, Thorax- und Gefäßchirurgie, Universitätsklinikum Bonn, Bonn, Germany; 3 Kinderchirurgische Klinik und Poliklinik, Dr. von Haunersches Kinderspital, Ludwig-Maximilians-Universität, München, Germany

**Keywords:** Health services research, Gastrointestinal surgery, Inpatients, Hospital reimbursement, Paediatric surgery, Population-based

## Abstract

**Purpose:**

Some paediatric surgical diseases showed a declining incidence in recent years, among which hypertrophic pyloric stenosis has been particularly striking shortly in the years after the millennium. We aimed to assess whether this development continued over the following decade, as it might offer the chance to better understand the underlying reasons.

**Methods:**

We analysed data files obtained from the German federal statistics office for principal diagnosis of hypertrophic pyloric stenosis and pyloromyotomies from 2005 to 2017. Changes over time were assessed via linear regression for incidences per 1000 live births.

**Results:**

In the respective time interval, there were a mean of 1009 pyloromyotomies (95% CI 906–1112) per year, of which a mean of 835 (95% CI 752–917) were performed in boys. The incidence of hypertrophic pyloric stenosis per 1000 live births almost halved between 2005 and 2017: it decreased by 0.12 pylorotomies annually (95% CI 0.09–0.14; *P* < 0.0001) in boys—from a maximum of 2.96 to a minimum of 1.63–and 0.03 pyloromyotomies annually (95% CI 0.02–0.04; *P* < 0.0001) in girls—from a maximum of 0.64 to a minimum of 0.28. There was considerable regional variation in incidences between the German länder.

**Conclusion:**

The decreasing incidence of hypertrophic pyloric stenosis noted around the millenium continued into the following decades. The underlying reasons are unclear, which should prompt further research on the subject matter.

## Introduction

In recent years, infantile hypertrophic pyloric stenosis is diagnosed earlier and thus metabolic derangements occur less often [1], although this development has not been observed in low- and middle-income countries, such as South Africa [2]. An important aspect in the care is the underlying epidemiology. Reports in the 1990s described an increasing incidence of infantile hypertrophic pyloric stenosis in the preceding decades [3, 4]. Contrary to this finding, more recent studies found a decreasing incidence of infantile hypertrophic pyloric stenosis not just in Europe [5–7], but also in North America [8, 9], Asia [10], and Oceania [11].

However, this finding seems to be influenced by seasonal [12], regional [7, 13], ethnicity, and sex-specific effects [8]. It is, therefore, crucial to analyse population-based data including these aspects. We aimed to describe the dynamic changes in incidence of hypertrophic pyloric stenosis from 2005 through 2017 on the basis of German administrative data to further elucidate the epidemiology of infantile hypertrophic pyloric stenosis by sex and region over time.

## Methods

Datasets from the *Statistisches Bundesamt* (Federal Statistics Office) including principal diagnoses and procedures of the German Modification of the International Classification of Diseases—Version 10 for the years 2005 to 2017 were obtained. Cases with operation codes for infantile pyloromyotomy (OPS 5-432.0) and the principal diagnosis of hypertrophic pyloric stenosis (ICD-10-GM Q40.0) were analysed. Only cases occurring in the first year of life were included. These administrative datasets count cases, not patients. Consequently, a patient may be included twice in case of a referral from one hospital to another. To quantify these cases, we subtracted the number of pyloromyotomies from the number of diagnoses of a hypertrophic pyloric stenosis. The detailed properties and pitfalls of these administrative dataset have been described elsewhere [14].

Statistical analysis were conducted using R (RRID: SCR_001905) (version 3.5.3) and its generic stats4-package [15]. We analysed differences over time by least square linear regression, as it has been deemed suitable for these data [16–18]. The assumptions of linear regression—normality of residuals and homogeneity of variances—were tested via the Shapiro–Wilk test and the *F* test using the olsrr-package (version 0.5.3) [19]. Confidence intervals for point estimates were calculated using the *t* distribution via the Rmisc-package (version 1.5) [20] and relative differences between diagnoses and procedures were compared using Student’s *t* test. Incidences were calculated using the number of pyloromyotomies per 1000 live births in Germany. Shape data for the visualisation of regional differences in the incidences of hypertrophic pyloric stenosis between the *Bundesländer* (German federal states) were obtained from the *Bundesamt für Kartographie und Geodäsie* (Federal Agency for Cartography and Geodesy) [21].

## Results

**Fig. 1:**
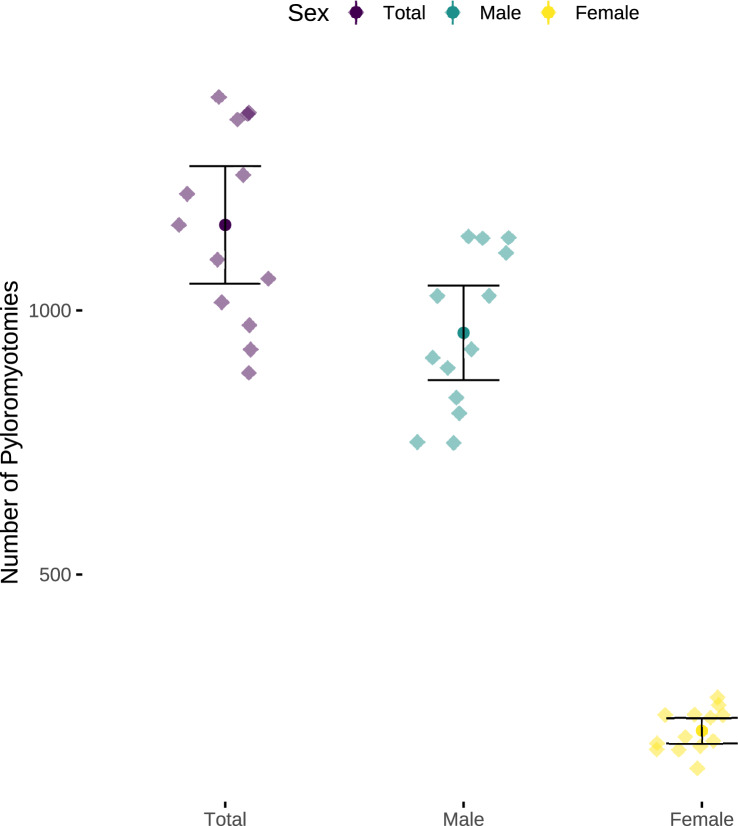
Pyloromyotomies per year. Data points represent individual yearly numbers of pyloromyotomies with mean and 95% confidence intervals

During the study period, a mean of 1009 [95% confidence interval (CI) 906–1112] pyloromyotomies was performed per year, of which 835 (95% CI 752–917) were in boys and 175 (95% CI 152–197) were in girls (Fig. 1). This resulted in a ratio of 4.8 cases in boys for every case in girls. The mean of live births corresponding to the pyloromyotomies was 701,293 (95% CI 674,890–727,696), of which $\overline{x}$ = 359,732 (95% CI 346,287–373,176) were boys and $\overline{x}$ = 341,559 (95% CI 328,600–354,519) were girls. This resulted in mean annual incidences were 2.34 (95% confidence interval 2.06–2.63) pyloromyotomies per 1000 live born boys and 0.52 (95% CI 0.44–0.6) per 1000 live born girls.

**Fig. 2:**
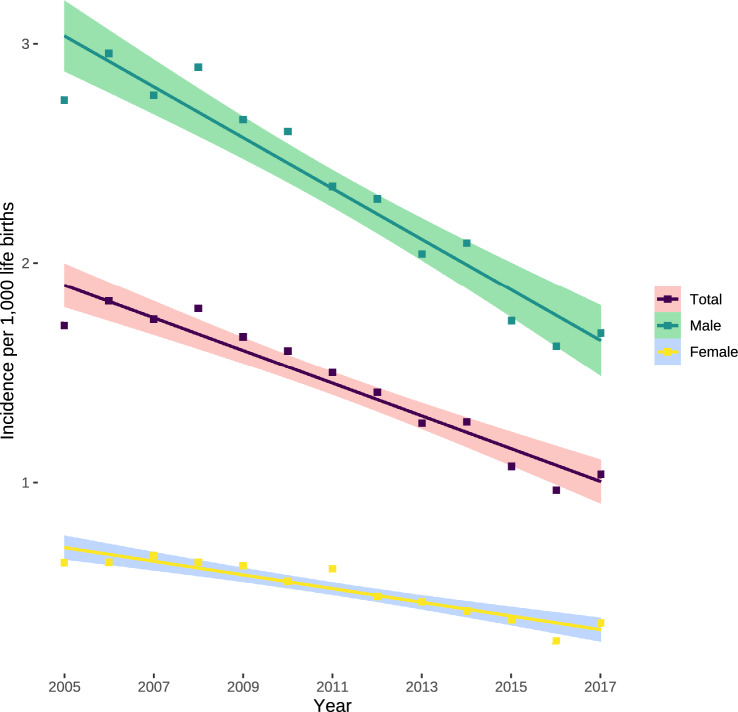
Incidence of pyloromyotomies per 1000 live births. Estimation is based on ordinary least square linear regression. Data are presented as slope with 95% confidence interval

Incidences decreased by 0.12 (95% CI 0.09–0.14; *P* < 0.0001) pyloromyotomies per 1000 male live births per year from the maximum of 2.96 in 2006 to a minimum of 1.62 in 2016 (Fig. 2). This decrease was even more pronounced in girls with a yearly decrease of 0.03 (95% CI 0.02–0.04; *P* < 0.0001) pyloromyotomies per 1000 female live births from a maximum of 0.64 in 2007 to a minimum of 0.28 in 2016 (Fig. 2).

The relative difference between cases diagnosed with hypertrophic pyloric stenosis and cases that underwent pyloromyotomy was 12.9% (95% confidence interval 12–13.9) in boys and 14.8% (95% confidence interval 12.2–17.5) in girls, indicating that a little over a tenth of the patients were transferred from one hospital to the other. Both relative differences did not change over time (♂: Δ = 0.18% (95% CI − 0.05 to 0.4), *P* = 0.1158; ♀: Δ = 0.6% (95% CI − 0.09 to 1.2), *P* = 0.0861) in the study period.

**Fig. 3:**
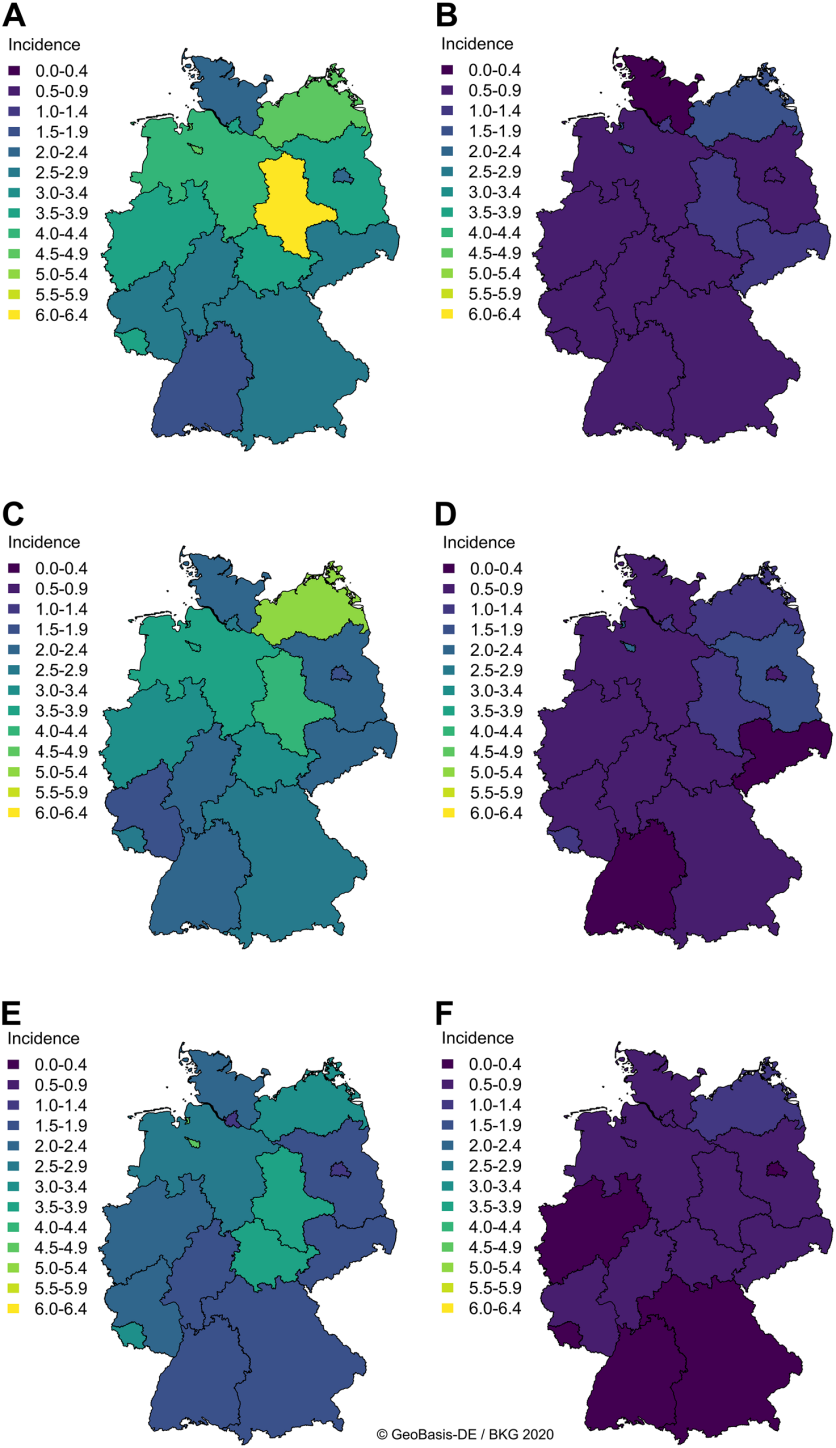
Regional variation in incidences of hypertrophic pyloric stenosis between the *Bundesländer* (German federal states). Data represent incidences of infantile hypertrophic pyloric stenosis per 1000 live born children in the German federal states in 2005 (**a, b**), 2011 (**c, d**), and 2017 (**e, f**) separated between males (**a, c, e**) and females (**b, d, f**)

We found considerable regional differences in incidences of infantile hypertrophic pyloric stenosis between the German federal states: mean incidences per 1000 live born boys varied from 1.95 (95% confidence interval 1.79–2.11) in Baden-Württemberg to 5.09 (95% CI 3.94–6.23) in Bremen (Table 1)—with a maximum observed in 2007 with 8.73 per 1000 live born boys *ibid* and a minimum of 1.05 per 1000 boys born alive 2016 in Berlin. Likewise, the highest incidence per 1000 live born girls was recorded in Bremen with a mean of 1.51 (95% CI 0.96–2.06), whereas the lowest mean incidence was found in Berlin with 0.5 (95% CI 0.38–0.61) per 1000 girls born alive (Table 1)—the maximum was observed in Bremen in 2010 with 3.3 cases per 1000 live born girls and the minimum with 0 cases per German federal state was also in Bremen in 2015 and the Saarland in 2017 with 3168 girls born alive in Bremen and 4047 in the Saarland. These regional differences persisted throughout the study duration despite the reduction in incidences (Fig. 3).

**Table 1:** Mean incidences and 95% confidence intervals of infantile hypertrophic pyloric stenosis per 1000 live births in boys and girls in the *Bundesländer* (German federal states)

German federal state	$\overline{X}$ ♂-incidence	95% CI	$\overline{X}$ ♀-incidence	95% CI
Baden-Württemberg	1.95	1.79–2.11	0.51	0.41–0.61
Bavaria	2.54	2.26–2.83	0.7	0.58–0.78
Berlin	1.99	1.59–2.39	0.5	0.38–0.61
Brandenburg	3.11	2.51–3.71	0.82	0.55–1.09
Bremen	5.09	3.94–6.23	1.51	0.96–2.06
Hamburg	3.1	2.23–3.97	0.73	0.51–0.94
Hesse	2.28	1.89–2.66	0.56	0.42–0.7
Mecklenburg Western Pomerania	4.32	3.78–4.87	0.92	0.57–1.26
Lower Saxony	3.28	2.81–3.74	0.78	0.67–0.89
Northrhine-Westphalia	3.19	2.79–3.59	0.83	0.7–0.96
Rhineland Palatinate	2.5	2.22–2.78	0.63	0.5–0.75
Saarland	4.3	3.27–5.29	1.03	0.74–1.32
Saxony	2.74	2.25–3.23	0.76	0.55–0.96
Saxony-Anhalt	4.32	3.5–5.14	1.33	0.92–1.74
Schleswig-Holstein	2.41	2.04–2.78	0.69	0.53–0.85
Thuringia	3.4	2.89–3.91	0.86	0.69–1.02

*♂* boys, *♀* girls, *CI* confidence interval

## Discussion

Studies from the twentieth century described an increasing incidence of hypertrophic pyloric stenosis [3, 4], whereas newer reports around the millennium documented a reversal of this trend [5–11]. A similar development has been described for other surgical diseases such as inguinal hernia as well [22–24]. The underlying reasons remain unclear throughout the reports, irrespective of the disease [8, 9, 11, 22, 23].

In a previous report from Germany, population-based data were assessed using only the number of coded ICD-10-GM diagnoses of infantile hypertrophic pyloric stenosis to describe the reduction in cases [7]. As the number of paediatric surgeons [25] and paediatric surgical units [26] has increased substantially since then, counting patients twice due to referral from one hospital to the other may have influenced the results. However, the small difference between diagnoses and procedures remained almost constant during the study period and is thus unlikely to have exerted a relevant influence on the results.

A previous study showed that the incidence of hypertrophic pyloric stenosis declined of by more than a third around the millennium [7]. Our study adds that this trend continued and perhaps even accelerated throughout our study period. Although cases may have been diagnosed earlier with less metabolic derangements than before in recent years [1], it is improbable that a case of hypertrophic pyloric stenosis would be managed entirely in an office-based outpatient setting and thus not appear among our data based on hospital reimbursements.

The ratio between male and female cases was calculated at 4.8:1 in our dataset—similar to preceding studies from Taiwan at 4.3:1 [10], 4.2:1 in Sweden [5], and 5.2:1 in the United States [27]. Some suggest that the reduction in hypertrophic pyloric stenosis might be caused by increasing maternal age in Western countries, and that boys may be particularly affected by this effect [9]. However, some regions in Europe recorded increasing incidences of hypertrophic pyloric stenosis despite increasing maternal age [13]. In addition, bottle-feeding has been associated with higher risk of developing hypertrophic pyloric stenosis [10], but adjustment for bottle-feeding did results in higher maternal age being highly associated with infantile hypertrophic pyloric stenosis [27]. Taken together, none of these factors was decisive and able to explain the decline. Nor have any of these factors been causally associated with the decrease in incidence of the other mentioned paediatric surgical diseases [22–24].

Similar to preceding reports [7, 13], we found relevant regional differences. Contrary to other regions in Europe [13], these differences between the German federal states were stable throughout the study period. A relevant variation could only be seen within German federal states with a small population and thus a low number of live births, in which random variation between the years could easily distort the numbers. Again, we may only describe this difference, but administrative data lack the necessary detail to explore the underlying effects due to their focus on outcome-related data.

In conclusion, the previously described substantial decline in the incidence of hypertrophic pyloric stenosis in Germany continues, although the reasons remain cryptic. However, administrative data are not suitable to explore associations and potentially causative factors. Consequently, future studies should focus on a multivariate analysis of possible associated factors: which underlying parameters changed over time and which remained stable? Administrative data cannot answer these questions.

## Data Availability

The data supporting the findings of this study are available from the Statistisches Bundesamt (German federal statistics office).
